# Effectiveness of Mobile App Interventions to Improve Periodontal Health: Protocol for a Systematic Review and Meta-Analysis

**DOI:** 10.2196/50479

**Published:** 2024-07-31

**Authors:** Reem Musa, Dalia Elamin, Robert Barrie, Faheema Kimmie-Dhansay

**Affiliations:** 1 Department of Community Dentistry Faculty of Dentistry University of Western Cape Cape Town South Africa

**Keywords:** mobile app, periodontal health, text messages, application, effectiveness, physical well-being, mental well-being, social well-being, oral hygiene, oral disease, disease prevention, periodontal, health education, systematic review

## Abstract

**Background:**

Periodontal health plays a key role as a shared reference point for evaluating periodontal diseases and identifying significant treatment outcomes. Providing adequate instruction and enhancing the motivation of patients to maintain proper oral hygiene are crucial factors for successful periodontal treatment, with self-performed regular oral hygiene identified as a critical factor in improving the outcomes of treatment for periodontal diseases. Recently, mobile health (mHealth) solutions, especially mobile apps, have emerged as valuable tools for self-management in chronic diseases such as periodontal disease, providing essential health education and monitoring capabilities. However, the use of mHealth apps for periodontal health is complex owing to various interacting components such as patient behavior, socioeconomic status, and adherence to oral hygiene practices. Existing literature has indicated positive effects of mHealth on oral health behaviors, knowledge, attitude, practice, plaque index score, and gingivitis reduction. However, there has been no systematic review of mobile apps specifically targeting patients with periodontal disease. Understanding the design and impact of mHealth apps is crucial for creating high-quality apps.

**Objective:**

The aim of this systematic review and meta-analysis is to evaluate the effectiveness of existing mobile apps in promoting periodontal health.

**Methods:**

A comprehensive search strategy will be performed in multiple electronic databases (PubMed, EBSCOhost, CINAHL Plus, Dentistry & Oral Sciences, ScienceDirect, Scopus, and Cochrane Central Register of Controlled Trials) with the following keywords in the title/abstract: “mobile application,” “mobile health,” “mHealth,” “telemedicine,” “periodontal health,” “periodontitis,” and “text message.” Only randomized controlled trials will be included that assessed the following outcomes to measure periodontal health improvement: gingival index, bleeding index, periodontal pocket depth, and clinical attachment loss. Covidence will be used for data collection, and a PRISMA (Preferred Reporting Items for Systematic reviews and Meta-Analyses) flowchart will be used to describe the selection process of the included, identified, and excluded studies. The Confidence in Network Meta-Analysis approach will be used for meta-analysis of the extracted data from the included studies.

**Results:**

This review will not require ethical approval since no primary data will be included. As of July 2024, a total of 83 articles retrieved from various databases have been imported to Covidence with 13 articles deemed eligible for inclusion in the review. The review is currently ongoing and is expected to be complete by the end of 2024 with the results published in early 2025.

**Conclusions:**

This systematic review and meta-analysis will contribute to developing mobile apps with enhanced criteria to improve periodontal clinical outcomes. The review emphasizes the importance of mHealth and preventing periodontal disease, which can set the stage for informed global health care strategies.

**Trial Registration:**

PROSPERO CRD42022340827; https://www.crd.york.ac.uk/prospero/display_record.php?RecordID=340827

**International Registered Report Identifier (IRRID):**

DERR1-10.2196/50479

## Introduction

### Background

Periodontal health is essential to overall oral health and well-being [1]. Periodontal health is generally defined as the absence of inflammatory periodontal disease [2], encompassing the health of the surrounding support system for the tooth, which includes the gingiva, periodontal ligament, and alveolar bone [3]. A standard definition of periodontal health is crucial for identifying a reference point to determine and assess treatment outcomes [2]. Building upon this understanding, periodontal health is classified into the following 4 levels, as proposed by Lang and Bartold [2], based on various factors such as the condition of the periodontium, the ability to control modifying factors, predisposing factors, and treatment outcomes:

1. Pristine periodontal health, representing the absence of clinical inflammation, usually with no bleeding on probing, bone loss, or attachment loss involvement, making it difficult to measure clinically.
2. Clinical periodontal health (intact periodontium), referring to minimal or absent gingival inflammation, which is clinically detectable with bleeding on probing.
3. Periodontal disease with stability in the reduced periodontium, defined as major plaque-associated periodontal diseases such as periodontitis, with the condition being effectively treated and no apparent worsening of clinical signs despite a reduced periodontium.
4. Periodontal disease in remission or under control, characterized by a reduction in the severity of symptoms, although they may not be resolved entirely.

Furthermore, the two primary periodontal conditions, gingivitis and periodontitis, play significant roles in oral health. Gingivitis, the mildest form of periodontal disease, is characterized by localized, reversible inflammation of the gingiva caused by dental plaque [4], whereas periodontitis is a microbe-associated, host-mediated inflammatory condition leading to progressive attachment loss [5]. Chronic conditions such as periodontal disease and dental caries can cause severe pain in advanced stages and may result in challenges with speaking and eating [6]. Additionally, there is a strong association between oral diseases and noncommunicable conditions such as diabetes [7] and cardiovascular disease [8].

In response to the evolving understanding of periodontal diseases, a global workshop was convened in 2017 to develop a new classification system to simplify diagnosing and treating these diseases in general dental practice [9]. This updated classification system addresses the form, severity, and extent of periodontitis and formally incorporates recognized risk factors and their associations with systemic diseases. Critical components of this classification system include staging, which categorizes the severity and extent of the disease, and grading, which predicts future risks and potential health impacts. This classification system allows for precise and focused definitions of periodontal health and gingivitis as follows: (1) patients with an intact periodontium, (2) patients with a reduced periodontium due to causes other than periodontitis, and (3) patients with a reduced periodontium due to periodontitis [9].

The previous classification was formally introduced in 1999 [10]; the notable distinction between the two classifications was the use of the term “periodontitis” instead of “aggressive and chronic periodontitis” [9]. Furthermore, smoking and diabetes were identified as major potential risk factors that could influence the staging of periodontal disease in the new classification [9].

As part of an accurate diagnosis and appropriate treatment strategy, having clear and focused definitions in a classification system is fundamental in promoting periodontal health. This understanding is vital as neglecting periodontal health can lead to significant conditions such as periodontal disease, bone loss, and ultimately tooth loss [10]. A combination of effective oral hygiene practices, routine dental examinations, and a healthy lifestyle is essential to maintain optimal periodontal health. Daily brushing and flossing help eliminate plaque, while professional scaling can remove calculus buildup [1]. The importance of effective self-performed oral hygiene in preventing oral diseases is well-established [11], including the integration of mobile health (mHealth) interventions to promote the self-management of oral health [12].

Indeed, the combination of mHealth technology in periodontal care has significantly impacted the field, offering a promising avenue for promoting oral health awareness and empowering patients to take control of their periodontal health [13,14]. mHealth is a medical and public health practice supported by mobile devices such as mobile phones, patient monitoring devices, personal digital assistants, and other wireless devices [15]. These mobile apps gather users’ health data, offer customized guidance, and facilitate remote patient data monitoring, thereby fostering collaborative relationships between dental professionals and individuals [16]. The increasing interest in chronic disease management has created opportunities to enhance self-management capabilities [17].

In light of these developments, Toniazzo et al [12] performed a systematic review to summarize the role of mHealth in improving oral health, with the measured outcomes being the plaque index and gingival index (GI). The review findings demonstrated a significant improvement in dental plaque and gingivitis resulting from the use of mobile apps, suggesting that these tools may enhance oral health behaviors. However, the lack of regulation during app development increases the risk of patients accessing inaccurate information [18]. Therefore, this review is designed as a preliminary step prior to the development of an mHealth-promotion app. To our knowledge, no other systematic review has been performed to specifically evaluate mobile apps targeting patients with periodontal disease.

Overall, integrating mHealth technology in periodontal care presents exciting opportunities for advancing oral health promotion and disease management. By embracing these innovations, dental professionals can enhance patient care and improve oral health outcomes while maintaining a comprehensive understanding of periodontal diseases.

### Review Objective

The aim of this systematic review and meta-analysis is to evaluate the effectiveness of existing mobile apps or text messages designed to enhance periodontal health among patients compared to the outcomes of patients without any mobile app or digital intervention to support their treatment.

## Methods

### Study Registration and Framework

This systematic review has been registered in PROSPERO under registration number CRD42022340827.

The Participants, Interventions, Comparators, Outcomes, and Study design (PICOS) framework is used to guide the eligibility criteria [19,20]. *Participants* include all patients, regardless of age or sex, with or without periodontal disease, except those who cannot participate independently. The *intervention* of interest is the use of mobile apps available on Android or iOS devices and mobile phones for sending SMS text messages or web-based interventions. As *comparators*, this review considers studies that compare the intervention to standard or usual care such as a placebo or control group without using mobile technology. The main *outcome* is improvement in the periodontal health of participants based on signs of progress in the following indicators: GI, bleeding index (BI), clinical attachment loss (CAL), and periodontal probing depth (PPD). All of these periodontal clinical parameters (described in further detail below) are considered crucial for assessing periodontal health because they provide valuable information about the condition of the gingiva and surrounding tissues [2].

### Outcome Measures

#### Gingival Index

The GI is a clinical examination tool used to assess the condition of the gingival tissue and differentiate the lesion severity. To perform the examination, the alterations in 4 areas around the tooth are located. Each area (mesial, distal, buccal, palatine, or lingual) is assigned a score ranging from 0 to 3. The overall score is calculated by summing the scores from the 4 areas and dividing the total by 4. [21]: a total score of 0 indicates a normal gingiva; a score of 1 represents mild inflammation, characterized by a slight color change and edema with no bleeding on probing; a score of 2 signifies moderate inflammation, characterized by redness and edema; and a score of 3 indicates severe inflammation marked by pronounced redness, edema, ulceration, and a tendency toward spontaneous bleeding [21,22].

#### Bleeding Index

The BI is a tool to assess and reflect the histological and bacterial factors associated with the severity of periodontal diseases [23]. The BI is typically determined using a Williams periodontal probe inserted into the gingival crevice to a depth of approximately 2 millimeters. The probe is angled at approximately 60° to the tooth’s longitudinal axis. The presence or absence of bleeding is then assessed within 30 seconds after probing [24].

#### Clinical Attachment Loss

CAL is one of the most important tools to measure periodontitis, which is crucial in diagnosing and managing periodontal diseases. CAL is calculated as the distance from the cementoenamel junction or the border of restoration to the bottom of the periodontal pocket [25].

#### Periodontal Probing Depth

PPD represents a deepened gingival sulcus around a tooth at the gingival margin due to pathological causes. The PPD is an important marker to indicate the presence of inflammation or infection and to monitor the progress of periodontal treatment [26]. The values are recorded in millimeters at 6 sites per tooth (mesio-buccal, mid-buccal, disto-buccal, mesio-lingual, mid-lingual, and disto-lingual) to measure the distance between the base of the pocket and the gingival margin [25].

### Inclusion and Exclusion Criteria

All published randomized controlled trials (RCTs) published in English will be included in the review. Studies aimed at improving tooth mobility and the grey literature will be excluded from this review. There will be no limitations on the time frame for measuring the outcome of the intervention.

### Search Strategy for Identification of Potential Studies

The search strategy for identifying potential studies will be designed by 3 team members (RM, DE, and FKD) to ensure a comprehensive exploration of eligible studies. This will involve performing a thorough search across various electronic databases, including PubMed, EBSCOhost, Dentistry & Oral Sciences, ScienceDirect, CINAHL Plus, and Scopus. The search will use combinations of Medical Subject Headings (MeSH) key terms in the title or abstract, including “mobile applications,” “periodontal health,” “periodontitis,” “mHealth,” “smartphone application,” and “app-based intervention.” These terms will be combined using Boolean operators such as “AND” and “OR” to capture relevant studies. Additionally, databases such as the Cochrane Central Register of Controlled Trials, International Clinical Trials Registry Platform [27], and ClinicalTrials.gov [28] will be searched. A final rerun of the search will be performed before the final meta-analysis to include any additional studies. An example of the search strategy for PubMed is provided in Multimedia Appendix 1.

### Study Selection Process

Two independent reviewers (RM and DE) will review all included articles. The selection will be based on the eligibility criteria and discrepancies will be resolved through consultation with a third reviewer (FKD). The identified articles will be imported to Covidence software [29], which is a web software system that assesses the data management process by reviewers during the systematic review process. The screening process will be performed independently by the two reviewers in 2 stages: (1) screening the abstract and (2) screening the full texts. Duplicate records will be removed automatically by Covidence.

Ongoing trials with no results will be flagged and recorded in an “Ongoing studies” table. Articles not meeting the eligibility criteria will be excluded and listed in a “Characteristics of excluded studies” table along with the reasons for exclusion. The study selection process will be described in a PRISMA (Preferred Reporting Items for Systematic reviews and Meta-Analyses) flowchart [30]. This protocol adheres to the PRISMA Protocols checklist (see Multimedia Appendix 2).

### Data Extraction and Management

Two independent reviewers (RM and DE) will extract data into Covidence using a specific form designed for this purpose. Data categories to be extracted include the field of research, sample size, setting, authors, publication year, countries, number of participants, app name and platform, app purpose, intervention period, and outcomes. Any disagreements will be resolved through discussion between the two reviewers. In cases where disagreements are unresolved, the third reviewer (FKD) will be consulted to reach a consensus through discussion.

### Assessment of Risk of Bias of Included Studies

Two independent reviewers (RM and DE) will assess the methodological quality of the included RCTs using the Cochrane Risk of Bias tool [31]. Any disagreements with quality ratings will be settled by consensus-based discussion.

The following domains for the risk of bias will be assessed for *all* included studies: random sequence generation (selection bias); allocation concealment (selection bias); blinding (performance bias and detection bias), separated for blinding of participants and personnel and blinding of outcome assessment; incomplete outcome data (attrition bias); selective reporting (reporting bias); and other bias [31].

The risk of bias for each domain will be categorized as high, low, or unclear. The risk of bias assessment outcomes will be documented in a table with a justification of the decision. A description of the criteria for a decision of “low risk,” “high risk,” or “unclear risk” will be documented [31].

### Meta-Analysis

#### Measures of Treatment Effect

If there is a probability that data are skewed with a fixed upper and lower limit, a “rule of thumb” calculation will be used to detect the distribution of the data. This calculation will follow the method proposed by Higgins et al [31], who suggested that if the SD doubled is greater than the mean, the assumption that the data are normally distributed cannot be made (ie, that the mean is at the center of the distribution). If the outcomes are dichotomous data, the data will be presented as risk ratios, odds ratios, or risk difference values. Data that include continuous outcomes will be presented as means or standardized mean differences. All significance tests will be judged at the 5% α level with 95% CIs. Time-to-event outcomes will be reported as hazard ratios and 95% CIs [31].

#### Assessment of Heterogeneity

The heterogeneity of results among studies will be interpreted by considering the variation of participant characteristics (eg, age) and trial characteristics such as randomization and allocation concealment in the different studies. Heterogeneity will be generally assessed by visually inspecting the forest plots to detect the proximity of the point estimates and the overlapping of CIs. Specifically, heterogeneity will be examined using the *I*² statistic with a *P* value threshold of <.10, which quantifies inconsistency across studies to assess its impact on the meta-analysis. The *I*^2^ statistic will be classified as follows: 0%-40%, negligible to minor heterogeneity; 30%-60%, moderate heterogeneity; 50%-90%, substantial heterogeneity; and 75%-100%, considerable heterogeneity.

Heterogeneity will further be investigated using sensitivity analysis. This involves assessing the robustness of the findings to different decisions or ranges of values made during the study. This process helps ascertain whether the findings are dependent on specific decisions or if they are robust and reliable [32,33]. If heterogeneity is found to be significant, the Mantel-Haenszel method will be used for the fixed-effects model. The random-effects model will be selected if *I*²≥50% or *P*<.10.

#### Assessment of Reporting Biases

When the outcomes are reported in 10 or more studies, a funnel plot will be constructed to investigate the possibility of reporting bias according to the guidelines outlined in the Cochrane Handbook for Systematic Reviews of Interventions [31].

#### Confidence of Study Findings

The results of the meta-analysis will be analyzed using the Confidence In Network Meta-Analysis (CINeMA) tool if there are more than 2 interventions included in the analysis; CINeMA is a software based on the framework developed by Salanti [34] and refined by Nikolakopoulou [35-37]. This framework’s efficiency lies in combining direct and indirect evidence from included studies in a meta-analysis when comparing more than one intervention.

Specifically, the CINeMA framework evaluates network meta-analysis through the following 6 domains: (1) within-study bias, (2) reporting bias, (3) indirectness, (4) imprecision, (5) heterogeneity, and (6) incoherence. The evaluation of each domain determines the level of concern, categorized as having no, some, or major concerns. These judgments across the domains are combined into a single confidence rating of “high,” “moderate,” “low,” or “very low.”

CINeMA serves as an alternative to the GRADE (Grading of Recommendations Assessment, Development, and Evaluation) approach, which has traditionally been used to assess confidence in the outcomes of systematic reviews and meta-analyses [33].

Data will be narratively synthesized when a meta-analysis is unsuitable according to the nature of included studies.

#### Sensitivity Analysis

A sensitivity analysis will be performed to determine the possible effect of loss to follow-up on the effect estimates for the primary outcomes. The results of the sensitivity analysis will be matched to the overall findings. However, for continuous data, the sensitivity analysis will be aligned with the methods described by Ebrahim et al [38,39]. These methods involve 5 sources of data for imputing means for participants with missing data: (1) best mean score among intervention arms, (2) best mean score among control arms, (3) mean score of the same trial among the control arms, (4) worst mean score among intervention arms, and (5) worst mean score among control arms [38].

### Dissemination of Findings and Data Sharing

All data, regardless of publication quality, will be included in the review. If study details cannot be obtained, the librarian will be consulted. If the study remains unobtainable, it will not be included in the meta-analysis.

## Results

Of the 159,214 publications identified, 83 were imported into Covidence to remove duplicates and perform the screening. This was followed by full-text reviewing and data extraction, resulting in 13 included articles. CINeMA analysis of the data is underway. This review is expected to be completed by the end of 2024, and we plan to publish the results in a peer-reviewed journal.

## Discussion

The significance of this systematic review and meta-analysis lies in its contribution to the development of mobile apps with enhanced criteria specifically aimed at improving periodontal clinical outcomes. To enhance the quality of new apps, a comprehensive understanding of the design and impact of mHealth apps is essential in improving treatment outcomes.

Using mHealth apps to improve periodontal health represents a complex intervention due to the numerous interacting components involved (eg, patient behaviors, socioeconomic status, and adherence to oral hygiene practices). The existing literature in this field has mainly focused on promoting oral health, hygiene, practices, and adherence to orthodontic treatment.

In a previous systematic review, Toniazzo et al [12] evaluated the effects of mobile apps on oral hygiene, revealing significant improvements in reducing dental plaque and gingivitis and in promoting better oral health behaviors. Another systematic review on the impact of digital media for promoting oral health showed improved knowledge, attitude, practice, and plaque index scores, along with overall gingivitis reduction [40].

Chen et al [41] performed a systematic review with a thematic focus on the prevention of dental caries through mobile apps aiming to improve oral hygiene, encourage adequate fluoride usage, and regulate dietary intake, while addressing adherence to orthodontic treatment. However, the authors concluded that dental caries outcomes management could have been more precise. In another systematic review and meta-analysis, Al-Moghrabi et al [42] assessed behavioral changes in orthodontic patients, covering aspects such as adherence to appliance use, oral health–related behaviors, hygiene levels, periodontal outcomes, appointment attendance, and knowledge, finding very low to moderate evidence supporting the effect of digital health; the authors thus recommended designing a well-constructed mobile app to enhance orthodontic outcomes.

Limiting our review to studies published in the English language may introduce bias. Nevertheless, we acknowledge that studies with significant findings are often published in English to enhance their visibility and citation potential. Furthermore, this review exclusively includes RCTs and we are excluding the grey literature. An important recommendation for future systematic reviews will be to assess the applicability and feasibility of the mHealth apps.

Finally, the broader implication of this systematic review and meta-analysis is its pivotal contribution to advancing the global integration of mobile apps for oral health by highlighting the positive impact of these tools on periodontal outcomes and emphasizing the importance of periodontal health management. These findings can help to enhance our understanding of mHealth interventions, elevate the quality of life among the target population, and set the stage for informed health care strategies in periodontal care.

## Appendix

Multimedia Appendix 1 Search strategy sample from PubMed.

Multimedia Appendix 2 PRIMSA-P (Preferred Reporting Items for Systematic reviews and Meta-Analyses Protocols) checklist.

## Abbreviations

BI: bleeding index
CAL: clinical attachment loss
CINeMA: Confidence In Network Meta-Analysis
GI: gingival index
GRADE: Grading of Recommendations Assessment, Development, and Evaluation
MeSH: Medical Subject Heading
mHealth: mobile health
PICOS: Participants, Interventions, Comparators, Outcomes, and Study design
PPD: periodontal pocket depth
PRISMA: Preferred Reporting Items for Systematic reviews and Meta-Analyses
RCT: randomized controlled trial
