# Supplementary material for: Effectiveness of Mobile App Interventions to Improve Periodontal Health: Protocol for a Systematic Review and Meta-Analysis
Source: JMIR Res Protoc. 2024 Jul 31;13:e50479. doi: 10.2196/50479 (PMC11325127; doi:10.2196/50479)
Supplement: Multimedia Appendix 1 [file resprot_v13i1e50479_app1.docx]

Multimedia Appendix 1. Search Strategy for PubMed

| **#** | **Search** | **Result** |
| --- | --- | --- |
| #1 | Mobile application (text word) | 1567 |
| #2 | Mobile application* | 1567 |
| #3 | Mobile applications (Mesh word) | 1324 |
| #4 | mHealth (text word) | 3536 |
| #5 | Telemedicine (Mesh word) | 3042 |
| #6 | Smart phones (text word) | 1312 |
| #7 | Smartphone (Mesh words) | 1365 |
| #8 | Text messages | 1469 |
| #9 | Text messaging | 1257 |
| #10 | Periodontal health (Text word) | 912 |
| #11 | Periodontitis (Mesh word) | 2710 |
| #12 | Periodontal disease (text word) | 2605 |
| #13 | Periodontal diseases (Mesh word) | 2544 |
| #14 | Gingivitis | 1894 |
| #15 | ((mobile application) OR (mobile applications)) OR (mobile application*) | 1567 |
| #16 | ((((((((mobile application) OR (mobile applications)) OR (mobile application*)) OR (mHealth)) OR (telemedicine)) OR (smart phones)) OR (smartphone) OR (text messages)) OR (text messaging) | 6075 |
| #17 | ((((periodontal health) OR (periodontal diseases)) OR (periodontal disease)) OR (periodontitis)) OR (gingivitis) | 3742 |
| #18 | (((((((((mobile application) OR (mobile applications)) OR (mobile application*)) OR (mHealth)) OR (telemedicine)) OR (smart phones)) OR (smartphone) OR (text messages)) OR (text messaging)) AND (((((periodontal health) OR (periodontal diseases)) OR (periodontal disease)) OR (periodontitis)) OR (gingivitis) | 1901 |

| **#** | **Search** | **Result** |
| --- | --- | --- |
| #1 | ("mobile applications"[MeSH Terms] OR ("mobile"[All Fields] AND "applications"[All Fields]) OR "mobile applications"[All Fields] OR ("mobile"[All Fields] AND "application"[All Fields]) OR "mobile application"[All Fields]) AND ((y_10[Filter]) AND (clinicaltrial[Filter] OR randomizedcontrolledtrial[Filter])) | 1567 |
| #2 | (("mobile"[All Fields] OR "mobiles"[All Fields]) AND "application*"[All Fields]) AND ((y_10[Filter]) AND (clinicaltrial[Filter] OR randomizedcontrolledtrial[Filter])) | 1567 |
| #3 | ("mobile applications"[MeSH Terms] OR ("mobile"[All Fields] AND "applications"[All Fields]) OR "mobile applications"[All Fields]) AND ((y_10[Filter]) AND (clinicaltrial[Filter] OR randomizedcontrolledtrial[Filter])) | 1324 |
| #4 | ("mhealth s"[All Fields] OR "telemedicine"[MeSH Terms] OR "telemedicine"[All Fields] OR "mhealth"[All Fields]) AND ((y_10[Filter]) AND (clinicaltrial[Filter] OR randomizedcontrolledtrial[Filter])) | 3536 |
| #5 | ("telemedicine"[MeSH Terms] OR "telemedicine"[All Fields] OR "telemedicine s"[All Fields]) AND ((y_10[Filter]) AND (clinicaltrial[Filter] OR randomizedcontrolledtrial[Filter])) | 3042 |
| #6 | ("smartphone"[MeSH Terms] OR "smartphone"[All Fields] OR ("smart"[All Fields] AND "phones"[All Fields]) OR "smart phones"[All Fields]) AND ((y_10[Filter]) AND (clinicaltrial[Filter] OR randomizedcontrolledtrial[Filter])) | 1312 |
| #7 | ("smartphone"[MeSH Terms] OR "smartphone"[All Fields] OR "smartphones"[All Fields] OR "smartphone s"[All Fields]) AND ((y_10[Filter]) AND (clinicaltrial[Filter] OR randomizedcontrolledtrial[Filter])) | 1365 |
| #8 | ("text messaging"[MeSH Terms] OR ("text"[All Fields] AND "messaging"[All Fields]) OR "text messaging"[All Fields] OR ("text"[All Fields] AND "messages"[All Fields]) OR "text messages"[All Fields]) AND ((y_10[Filter]) AND (clinicaltrial[Filter] OR randomizedcontrolledtrial[Filter]) | 1469 |
| #9 | ("text messaging"[MeSH Terms] OR ("text"[All Fields] AND "messaging"[All Fields]) OR "text messaging"[All Fields]) AND ((y_10[Filter]) AND (clinicaltrial[Filter] OR randomizedcontrolledtrial[Filter])) | 1257 |
| #10 | (("periodontal"[All Fields] OR "periodontally"[All Fields] OR "periodontically"[All Fields] OR "periodontics"[MeSH Terms] OR "periodontics"[All Fields] OR "periodontic"[All Fields] OR "periodontitis"[MeSH Terms] OR "periodontitis"[All Fields] OR "periodontitides"[All Fields]) AND ("health"[MeSH Terms] OR "health"[All Fields] OR "health s"[All Fields] OR "healthful"[All Fields] OR "healthfulness"[All Fields] OR "healths"[All Fields])) AND ((y_10[Filter]) AND (clinicaltrial[Filter] OR randomizedcontrolledtrial[Filter])) | 912 |
| #11 | ("periodontal"[All Fields] OR "periodontally"[All Fields] OR "periodontically"[All Fields] OR "periodontics"[MeSH Terms] OR "periodontics"[All Fields] OR "periodontic"[All Fields] OR "periodontitis"[MeSH Terms] OR "periodontitis"[All Fields] OR "periodontitides"[All Fields]) AND ((y_10[Filter]) AND (clinicaltrial[Filter] OR randomizedcontrolledtrial[Filter])) | 2710 |
| #12 | ("periodontal diseases"[MeSH Terms] OR ("periodontal"[All Fields] AND "diseases"[All Fields]) OR "periodontal diseases"[All Fields] OR ("periodontal"[All Fields] AND "disease"[All Fields]) OR "periodontal disease"[All Fields]) AND ((y_10[Filter]) AND (clinicaltrial[Filter] OR randomizedcontrolledtrial[Filter])) | 2605 |
| #13 | ("periodontal diseases"[MeSH Terms] OR ("periodontal"[All Fields] AND "diseases"[All Fields]) OR "periodontal diseases"[All Fields]) AND ((y_10[Filter]) AND (clinicaltrial[Filter] OR randomizedcontrolledtrial[Filter])) | 2544 |
| #14 | "gingiva"[MeSH Terms] OR "gingiva"[All Fields] OR "gingival"[All Fields] OR "gingivally"[All Fields] OR "gingivals"[All Fields] OR "gingivitis"[MeSH Terms] OR "gingivitis"[All Fields] OR "gingivitides"[All Fields]) AND ((y_10[Filter]) AND (clinicaltrial[Filter] OR randomizedcontrolledtrial[Filter])) | 1894 |
| #15 | ("mobile applications"[MeSH Terms] OR ("mobile"[All Fields] AND "applications"[All Fields]) OR "mobile applications"[All Fields] OR ("mobile"[All Fields] AND "application"[All Fields]) OR "mobile application"[All Fields] OR ("mobile applications"[MeSH Terms] OR ("mobile"[All Fields] AND "applications"[All Fields]) OR "mobile applications"[All Fields]) OR (("mobile"[All Fields] OR "mobiles"[All Fields]) AND "application*"[All Fields])) AND ((y_10[Filter]) AND (clinicaltrial[Filter] OR randomizedcontrolledtrial[Filter])) | 1567 |
| #16 | ("mobile applications"[MeSH Terms] OR ("mobile"[All Fields] AND "applications"[All Fields]) OR "mobile applications"[All Fields] OR ("mobile"[All Fields] AND "application"[All Fields]) OR "mobile application"[All Fields] OR ("mobile applications"[MeSH Terms] OR ("mobile"[All Fields] AND "applications"[All Fields]) OR "mobile applications"[All Fields]) OR (("mobile"[All Fields] OR "mobiles"[All Fields]) AND "application*"[All Fields]) OR ("mhealth s"[All Fields] OR "telemedicine"[MeSH Terms] OR "telemedicine"[All Fields] OR "mhealth"[All Fields]) OR ("telemedicine"[MeSH Terms] OR "telemedicine"[All Fields] OR "telemedicine s"[All Fields]) OR ("smartphone"[MeSH Terms] OR "smartphone"[All Fields] OR ("smart"[All Fields] AND "phones"[All Fields]) OR "smart phones"[All Fields]) OR ("smartphone"[MeSH Terms] OR "smartphone"[All Fields] OR "smartphones"[All Fields] OR "smartphone s"[All Fields])) AND ((y_10[Filter]) AND (clinicaltrial[Filter] OR randomizedcontrolledtrial[Filter])) | 6075 |
| #17 | ((("periodontal"[All Fields] OR "periodontally"[All Fields] OR "periodontically"[All Fields] OR "periodontics"[MeSH Terms] OR "periodontics"[All Fields] OR "periodontic"[All Fields] OR "periodontitis"[MeSH Terms] OR "periodontitis"[All Fields] OR "periodontitides"[All Fields]) AND ("health"[MeSH Terms] OR "health"[All Fields] OR "health s"[All Fields] OR "healthful"[All Fields] OR "healthfulness"[All Fields] OR "healths"[All Fields])) OR ("periodontal diseases"[MeSH Terms] OR ("periodontal"[All Fields] AND "diseases"[All Fields]) OR "periodontal diseases"[All Fields]) OR ("periodontal diseases"[MeSH Terms] OR ("periodontal"[All Fields] AND "diseases"[All Fields]) OR "periodontal diseases"[All Fields] OR ("periodontal"[All Fields] AND "disease"[All Fields]) OR "periodontal disease"[All Fields]) OR ("periodontal"[All Fields] OR "periodontally"[All Fields] OR "periodontically"[All Fields] OR "periodontics"[MeSH Terms] OR "periodontics"[All Fields] OR "periodontic"[All Fields] OR "periodontitis"[MeSH Terms] OR "periodontitis"[All Fields] OR "periodontitides"[All Fields]) OR ("gingiva"[MeSH Terms] OR "gingiva"[All Fields] OR "gingival"[All Fields] OR "gingivally"[All Fields] OR "gingivals"[All Fields] OR "gingivitis"[MeSH Terms] OR "gingivitis"[All Fields] OR "gingivitides"[All Fields])) AND ((y_10[Filter]) AND (clinicaltrial[Filter] OR randomizedcontrolledtrial[Filter])) | 3742 |
| #18 | ((("mobile applications"[MeSH Terms] OR ("mobile"[All Fields] AND "applications"[All Fields]) OR "mobile applications"[All Fields] OR ("mobile"[All Fields] AND "application"[All Fields]) OR "mobile application"[All Fields] OR ("mobile applications"[MeSH Terms] OR ("mobile"[All Fields] AND "applications"[All Fields]) OR "mobile applications"[All Fields]) OR (("mobile"[All Fields] OR "mobiles"[All Fields]) AND "application*"[All Fields]) OR ("mhealth s"[All Fields] OR "telemedicine"[MeSH Terms] OR "telemedicine"[All Fields] OR "mhealth"[All Fields]) OR ("telemedicine"[MeSH Terms] OR "telemedicine"[All Fields] OR "telemedicine s"[All Fields]) OR ("smartphone"[MeSH Terms] OR "smartphone"[All Fields] OR ("smart"[All Fields] AND "phones"[All Fields]) OR "smart phones"[All Fields]) OR ("smartphone"[MeSH Terms] OR "smartphone"[All Fields] OR "smartphones"[All Fields] OR "smartphone s"[All Fields]) OR ("text messaging"[MeSH Terms] OR ("text"[All Fields] AND "messaging"[All Fields]) OR "text messaging"[All Fields] OR ("text"[All Fields] AND "messages"[All Fields]) OR "text messages"[All Fields]) OR ("text messaging"[MeSH Terms] OR ("text"[All Fields] AND "messaging"[All Fields]) OR "text messaging"[All Fields])) AND ((("periodontal"[All Fields] OR "periodontally"[All Fields] OR "periodontically"[All Fields] OR "periodontics"[MeSH Terms] OR "periodontics"[All Fields] OR "periodontic"[All Fields] OR "periodontitis"[MeSH Terms] OR "periodontitis"[All Fields] OR "periodontitides"[All Fields]) AND ("health"[MeSH Terms] OR "health"[All Fields] OR "health s"[All Fields] OR "healthful"[All Fields] OR "healthfulness"[All Fields] OR "healths"[All Fields])) OR ("periodontal diseases"[MeSH Terms] OR ("periodontal"[All Fields] AND "diseases"[All Fields]) OR "periodontal diseases"[All Fields]) OR ("periodontal diseases"[MeSH Terms] OR ("periodontal"[All Fields] AND "diseases"[All Fields]) OR "periodontal diseases"[All Fields] OR ("periodontal"[All Fields] AND "disease"[All Fields]) OR "periodontal disease"[All Fields]) OR ("periodontal"[All Fields] OR "periodontally"[All Fields] OR "periodontically"[All Fields] OR "periodontics"[MeSH Terms] OR "periodontics"[All Fields] OR "periodontic"[All Fields] OR "periodontitis"[MeSH Terms] OR "periodontitis"[All Fields] OR "periodontitides"[All Fields]))) OR ("gingiva"[MeSH Terms] OR "gingiva"[All Fields] OR "gingival"[All Fields] OR "gingivally"[All Fields] OR "gingivals"[All Fields] OR "gingivitis"[MeSH Terms] OR "gingivitis"[All Fields] OR "gingivitides"[All Fields])) AND ((y_10[Filter]) AND (clinicaltrial[Filter] OR randomizedcontrolledtrial[Filter])) | 1901 |
